# Comparison of major bleeding in patients with acute coronary syndrome that underwent coronary artery bypass grafting treated with clopidogrel or ticagrelor: a systematic review and meta-analysis

**DOI:** 10.12688/f1000research.21925.2

**Published:** 2020-09-07

**Authors:** Mohammad Saifur Rohman, Yeni Purnamasari, Muhammad Ilmawan, Bagus Aulia Mahdi, Fredo Tamara, Aditya Indra Mahendra, Mazen Mazen, Teuku Heriansyah, Muhammad Yamin, Budi Susetio Pikir, Jonny Karunia Fajar

**Affiliations:** 1 Brawijaya Cardiovascular Research Center, Department of Cardiology and Vascular Medicine, Faculty of Medicine, Universitas Brawijaya, Malang, East Java, 65145, Indonesia; 2 Faculty of Medicine, Universitas Brawijaya, Malang, East Java, 65145, Indonesia; 3 Department of Internal Medicine, Faculty of Medicine, Universitas Airlangga, Surabaya, East Java, 60115, Indonesia; 4 Brawijaya Internal Medicine Research Center, Department of Internal Medicine, Faculty of Medicine, Universitas Brawijaya, Malang, East Java, 65145, Indonesia; 5 Department of Cardiology and Vascular Medicine, Faculty of Medicine, Universitas Syiah Kuala, Banda Aceh, Aceh, 23111, Indonesia; 6 Division of Cardiovascular Medicine, Department of Internal Medicine, Faculty of Medicine, Universitas Indonesia, Jakarta, Jakarta, 16424, Indonesia; 7 Department of Cardiology and Vascular Medicine, Faculty of Medicine, Universitas Airlangga, Surabaya, East Java, 60115, Indonesia

**Keywords:** major bleeding, coronary artery bypass grafting, clopidogrel, ticagrelor

## Abstract

**Background: **There is controversy among physicians regarding the use of dual antiplatelet therapy (DAPT) in acute coronary syndrome (ACS) patients treated with coronary artery bypass grafting (CABG). Moreover, the evidence of previous studies about this topic remained inconclusive. This study aimed to perform a meta-analysis concerning the relation between the risk of major bleeding and the use of different DAPT (clopidogrel or ticagrelor) in ACS patients treated with CABG.

**Methods:** A meta-analysis was conducted during March to October 2019. Searches were carried out in Pubmed, Embase, Cochrane, and Web of Science. The predictor covariate in our present study was DAPT (clopidogrel or ticagrelor), and the outcome measure was the risk of major bleeding. Sub-group analysis was also performed, where data were classified into pre- and post-CABG. Furthermore, to determine the correlation and effect estimation, data were analyzed using fixed or random effect model.

**Results:** A total of 13 studies consisting 34,015 patients treated with clopidogrel and 32,661 patients treated with ticagrelor was included in our study. Our pooled calculation revealed that the incidence of major bleeding was not different significantly between clopidogrel and ticagrelor. In pre- and post-CABG sub-groups, our results also found no significant difference in major bleeding incidence between clopidogrel and ticagrelor groups.

**Conclusions:** Our meta-analysis clarifies that clopidogrel, compared to ticagrelor, or vice versa, is not associated with the risk of major bleeding in ACS patients treated with CABG.

## Introduction

In the last two decades, the management of acute coronary syndrome (ACS) has been well defined and periodically updated. Management has developed drastically over this period ^1^. Management options are numerous, and they depend on the facilities of the hospital. Of these treatment options, coronary artery bypass grafting (CABG) is considered the most challenging and the final option when other treatment options, including percutaneous coronary intervention (PCI) and thrombolytic therapy, fail to restore blood flow in the infarct-related artery ^2^. Moreover, the drugs used in ACS patients in all management options are complex, and dual antiplatelet therapy (DAPT) is commonly used. DAPT is globally used to treat patients with ACS. It was first reported in 1996 ^3^, and was first recommended for treating ACS patients in 2007 in American College of Cardiology (ACC)/American Heart Association (AHA) guidelines ^4^. Since then, DAPT has been widely used in the early management of ACS patients ^5, 6^.

Recently, when performing DAPT, whether to use clopidogrel or ticagrelor (the choice between acetylsalicylic acid (ASA) + clopidogrel and ASA + ticagrelor) has remained controversial due to the current assumption that one of the two might provide higher risk of major bleeding ^7, 8^. In the Indonesian National Health Insurance drug catalog, in 2018 clopidogrel was withdrawn and substituted with ticagrelor. However, in the drug price list ( https://e-katalog.lkpp.go.id/; website in Indonesian), ticagrelor is more expensive than clopidogrel. It is unclear whether the assumptions made about the risk of major bleeding caused by clopidogrel or ticagrelor were supported by the evidence or were possibly the result of conspiracy among pharmaceutical industries to increase their products marketing. Ticagrelor may provide a more potent platelet inhibition effect, therefore reducing the risk of a thrombotic event ^9^. In the context of ACS, the greater effect may be accompanied more complications. Therefore, the benefits of DAPT and the risk of complications (bleeding) should balance. In the case of ACS patients undergoing CABG, to prevent major bleeding, it is recommended that DAPT should be discontinued for at least three and five days before elective CABG for ticagrelor and clopidogrel, respectively ^10^. Furthermore, in the case of emergency or urgent CABG, DAPT should be discontinued prematurely ^11^. The discontinuation of DAPT might increase the risk of a thrombotic event ^12^. However, delay in CABG had also been shown to associate with poor clinical outcome and increased risk of mortality ^13^. Therefore, identifying the appropriate DAPT, whether ticagrelor or clopidogrel, is crucial to prevent the risk of major bleeding. Although 2016 ACC/AHA guidelines recommended ticagrelor over clopidogrel because ticagrelor is considered to have a more potent anti-platelet effect than clopidogrel ^14^, the evidence from previous studies regarding the association between the risk of major bleeding and the use of different DAPT using either clopidogrel or ticagrelor in ACS patients treated with CABG were inconclusive. Therefore, those inconclusive data of previous studies required clarification using a meta-analysis approach.

Therefore, the present study aimed to perform a meta-analysis whether the use of different DAPT (clopidogrel or ticagrelor) might affect the risk of major bleeding or not in ACS patients treated with CABG. Our study outcome could clarify the real effect of the use of DAPT (clopidogrel or ticagrelor) to the risk of major bleeding in ACS patients treated with CABG. Moreover, we also expect that our current meta-analysis might correct previous assumptions concerning the use of different DAPT.

## Methods

### Study design

A Meta-analysis was performed during March to October 2019 to assess the association between the incidence of major bleeding and the use of DAPT either clopidogrel or ticagrelor in ACS patients treated with CABG. In effort to attain our goal, potentially relevant papers were identified and collected from PubMed, Embase, Cochrane, and Web of Science to calculate odd ratio (OR) and 95% confidence interval (95%CI) using either fixed or random effect model. A checklist adapted from Preferred Reporting Items for Systematic Review and Meta-Analysis (PRISMA) and the design of our previous meta-analyses ^15– 20^ were used to guide the meta-analysis protocols in our present study ^21^. See *Reporting guidelines* for a completed PRISMA checklist for this study ^22^.

### Search strategy

We conducted a systematic search in PubMed, Embase, Cochrane, and Web of Science up to 20 September 2019. The search strategy, conformed to medical subjects heading (MeSH), involved the use of combination the following keywords: ["Major Bleeding"] AND ["Coronary Artery Bypass Grafting" OR "CABG"] AND ["Dual Anti Platelet Therapy" OR "DAPT"], and ["Clopidogrel" OR "Ticagrelor"]. In our searching strategy, language restrictions were not applied. We only used the study with the larger sample size and that was more up-to-date if we found the same data among studies. Moreover, we also searched the potential papers from the reference list of relevant or eligible studies. We also employed the "related article" option in PubMed to broaden our searching strategy. The potentially relevant papers were identified by two independent investigators (Y.P., M.I.). Disagreement between two independent investigators was resolved by discussion and/or by consulting to the senior investigator (J.K.F.).

### Eligibility criteria and data extraction

The inclusion criteria for this study were (1) retrospective studies, (2) prospective studies, (3) randomized controlled trials (RCTs), (4) evaluating the association between the incidence of major bleeding and DAPT either using clopidogrel or ticagrelor in ACS patients treated with CABG, (5) providing sufficient data for calculation of OR with 95% CI. While, articles were excluded if the following criteria were found: (1) irrelevant topic, (2) review, (3) conference presentation, and (4) having low quality (see *Quality assessment*). For data extraction, information related to (1) name of the first author, (2) year of publication, (3) country of origin, (4) sample sizes of case and controls, and (5) the incidence of major bleeding were extracted from each study. To prevent human errors, data extraction was performed by two independent authors. If discrepancy occurred, a consensus or discussion was established.

### Covariates and sub-group analysis

The predictor covariate in this study was DAPT either using clopidogrel or ticagrelor. While, the main outcome measure was the incidence of major bleeding in patients receiving both clopidogrel and ticagrelor. The major bleeding included in our analysis was restricted to thrombolysis in myocardial infarction ^23^ and platelet inhibition and outcomes criteria ^24^. Moreover, to confer a comprehensive analysis, we also performed sub-group analysis. Data were classified into the incidence of major bleeding in ACS patients treated with DAPT (clopidogrel or ticagrelor) before and after CABG.

### Quality assessment

To ensure the quality of each study and to avoid the potential bias in each study, the quality of retrieved studies was controlled and collected by two independent investigators (Y.P., M.I.). The quality and risk of bias of each study was assessed using Methodological Index for Non-Randomized Studies (MINORS) score ^25^. The MINORS score ranged from 0 to 24, and consisted of 12 items. Each item was assessed as 0 if the item was not reported, 1 if the item was inadequate reported, and 2 if the item was adequate reported. Each study was interpreted as having low quality if the score was less than or equal to 12, moderate if the score was less than or equal to 16 and more than 12, and high quality if the score was more than 16 ^25^. If disagreement was found between two independent authors, consensus was achieved through discussion between the two investigators. If the disagreement was not resolved, a consultation to senior researcher (JKF) was conducted.

### Statistical analysis

The comparison and effect estimation of major bleeding between DAPT with clopidogrel and ticagrelor were determined using the Z-test. The pooled calculation and effect estimation were described using forest plots. The model of forest plot for describing the comparison and effect estimation was conformed with a Q test. Before analysis using the Z-test, we evaluated heterogeneity and potential publication bias. A Q-test was employed to evaluate heterogeneity. P-value of less than 0.10 was considered to indicate heterogeneity. If we found heterogeneity, a random effect model was used. While, if heterogeneity was not found, a fixed effect model was used. For testing publication bias, an Egger test was used. A P-value of less than 0.05 was considered significantly having publication bias. All analyses in our study were carried out using Review Manager version 5.3 (RevMan Cochrane, London, UK) and Comprehensive Meta-Analysis (CMA, New Jersey, US) version 2.1.

## Results

### Eligible studies

**Figure 1.**
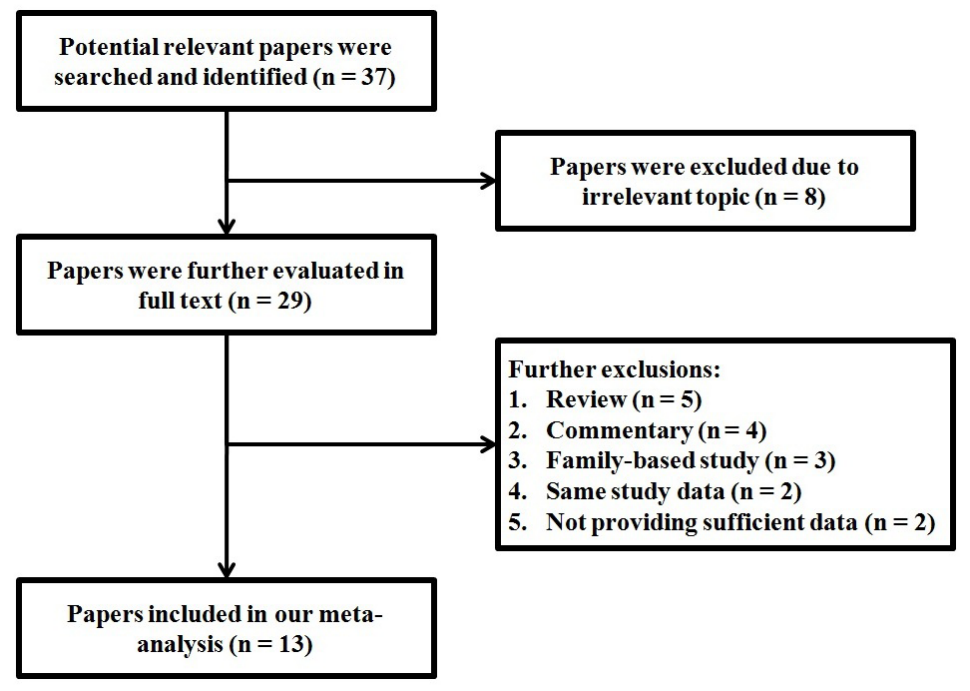
A Flowchart diagram of the article search and study selection.

A flowchart of article searches and study selection is shown in Figure 1. Initially, 37 articles were identified from the literature search. However, eight of them were excluded because they did not have relevance to the topic, leaving a total of 29 articles. The full text of these articles was retrieved and reviewed; it was found that 16 studies did not meet the eligibility criteria because they were reviews (n=5), commentaries (n=4), family-based studies (n=3), included the same study data (n=2), and not providing sufficient data for calculation of OR and 95%CI (n=2). Finally, a total of 13 studies were eligible for our meta-analysis. Baseline characteristics of studies included in our analysis are provided in Table 1.

**Table 1.** Baseline characteristics of studies included in the meta-analysis.

Author and year	Clopidogrel		Ticagrelor		Setting (before/after CABG)	Case	Study design	Ethnicity	Age (mean±SD)	Bleeding assessment	MINORS
	MB	n	MB	n							
Becker *et al.* 2011 ^26^	476	9186	446	9235	after CABG	STEMI & NSTEMI	RCT	Mixed	65.5±4.9	TIMI	24
Chang *et al.* 2019 ^2^	7	100	7	100	after CABG	NSTEMI	Case - control	Asian	64.0±10.0	TIMI	16
Dery *et al.* 2014 ^27^	37	328	6	55	after CABG	ACS	Case - control	Caucasian	65.5±2.2	BARC-CABG major bleeding	18
Dinicolantonio *et al.* 2013 ^28^	654	9186	619	9235	before CABG	ACS	Case - control	Mixed	64.5±2.2	TIMI	18
Gajayana *et al.* 2018 ^29^	44	1721	7	860	before CABG	ACS	Case - control	Caucasian	63.0±2.6	TIMI	17
Hansson *et al.* 2014 ^30^	213	232	166	173	before CABG	ACS	Case - control	Caucasian	67.0±9.6	TIMI	24
Hansson *et al.* 2016 ^31^	95	978	90	1266	before CABG	ACS	Case - control	Caucasian	67.5±9.5	BARC-CABG major bleeding	19
Held *et al.* 2011 ^32^	375	629	362	632	before CABG	ACS	Case - control	Caucasian	64.0±12.0	TIMI	24
Holm *et al.* 2019 ^33^	474	1293	381	1018	before CABG	ACS	Cohort	Mixed	66.3±9.6	PLATO	24
Kang *et al.* 2015 ^34^	929	9291	961	9332	after CABG	ACS	Case - control	Asian	61.3±9.0	PLATO	23
Russo *et al.* 2018 ^35^	19	413	5	95	before CABG	ACS	Case - control	Caucasian	66.7±13.0	BARC-CABG major bleeding	22
Schaefer *et al.* 2016 ^36^	0	28	2	28	before CABG	ACS	Case - control	Caucasian	73.0±6.4	TIMI	14
Varenhorst *et al.* 2012 ^37^	62	629	32	632	before CABG	ACS	RCT	Mixed	66.8±3.2	TIMI	24

MB, major bleeding; n, sample size; CABG, coronary artery bypass grafting; RCT, randomized controlled trial; MINORS, Methodological Index for Non-Randomized Studies; STEMI, ST-Elevation Myocardial Infarction; NSTEMI, Non-ST-elevation myocardial infarction; ACS, acute coronary syndrome; TIMI, Thrombolysis In Myocardial Infarction; BARC, Bleeding Academic Research Consortium; PLATO, Platelet inhibition and Outcomes.

### Data synthesis

**Figure 2.**
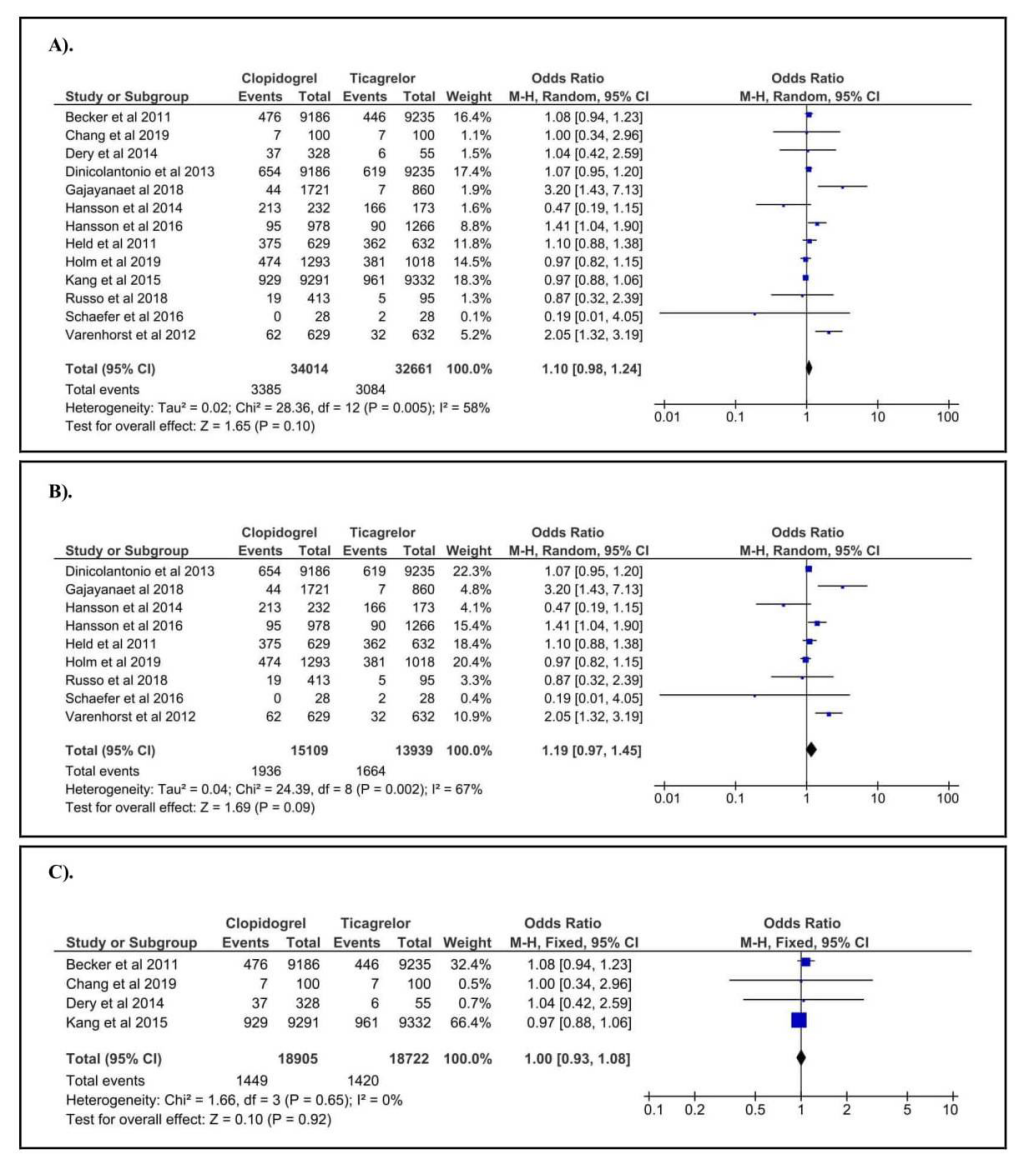
Forest plot of major bleeding comparison between clopidogrel and ticagrelor. ( **A**) Overall analysis. ( **B**) Pre-coronary artery bypass grafting (CABG) sub-group. ( **C**) Post-CABG sub-group.

A total of 13 studies ^2, 26– 37^, consisting 34,014 patients treated with clopidogrel and 32,661 patients treated with ticagrelor, were included in our study. Of those, the correlation between the use of DAPT (either clopidogrel or ticagrelor) and the risk of major bleeding was found in only three studies ^29, 30, 37^. A further ten studies failed to clarify the association ^2, 26– 28, 31– 36^. Our calculation revealed ( Figure 2A) that the incidence of major bleeding was not significantly different between clopidogrel and ticagrelor (OR = 1.10, 95%CI = 0.98–1.24, p = 0.0990). Moreover, in pre-CABG sub-group, we included nine studies ^28– 33, 35– 37^ consisting of 15,109 patients treated with clopidogrel and 13,939 patients treated with ticagrelor. Our results found ( Figure 2B) that no significant different of major bleeding incidence was observed between clopidogrel and ticagrelor (OR = 1.19, 95%CI = 0.97–1.45, p = 0.0910). While, in the post-CABG sub-group, a total of four papers ^2, 26, 27, 34^ consisting of 18,905 patients treated with clopidogrel and 18,722 patients treated with ticagrelor was enrolled for our analysis. Our pooled data ( Figure 2C) confirmed no significant different in major bleeding incidence between clopidogrel and ticagrelor (OR = 1.00, 95%CI = 0.93–1.08, p = 0.9230). The summary of correlation and effect estimation between the risk of major bleeding and the use of different DAPT is provided in Table 2.

**Table 2.** Summary of the association between the use of different dual antiplatelet therapies and the risk of major bleeding.

Parameters	Clopidogrel		Ticagrelor		Model	OR	95%CI	pHet	pE	P-value
	MB, n [%]	Patients, n	MB, n [%]	Patients, n						
Overall analysis	3,385 [9.95]	34,014	3084 [9.44]	32,661	Random	1.10	0.98–1.24	0.0050	0.1310	0.0990
Pre-CABG sub-group	1,936 [12.81]	15,109	1664 [11.94]	13,939	Random	1.19	0.97–1.45	0.0020	0.2060	0.0910
Post-CABG sub-group	1,449 [7.66]	18,905	1,420 [7.58]	18,722	Fixed	1.00	0.93–1.08	0.6470	<0.0001	0.9230

CABG, coronary artery bypass grafting; MB, major bleeding; OR, odds ratio; CI, confidence interval; pHet, p heterogeneity; pE, p Egger.

### Heterogeneity and publication bias

Evidence of heterogeneity was assessed using the Q-test. Our analysis found that evidence of heterogeneity (p <0.10) was observed in overall analysis and pre-CABG sub-group. Therefore, random effect model was applied to determine the correlation and effect estimation. While, for post-CABG sub-group, we used fixed effect model to assess the correlation and effect estimation because we did not find the evidence of heterogeneity. Furthermore, potential publication bias was assessed using an Egger test. Our analysis confirmed that potential publication bias was found in post-CABG sub-group (p <0.05). In overall analysis and pre-CABG sub-group, we found no publication bias. The summary of study heterogeneity and potential publication is described in Table 2.

## Discussion

Our current findings confirmed that neither clopidogrel nor ticagrelor was associated with risk of major bleeding among ACS patients treated with CABG. To our knowledge, no previous meta-analysis has reported the comparison between clopidogrel and ticagrelor in the context of CABG. Therefore, we were unable to perform a direct comparison. However, in other case settings, meta-analyses have been conducted in the case of PCI and thrombolytic for treating ACS patients. In the case of PCI for treating ACS patients, the reports from previous meta-analyses remained conflicting. A study conducted by Fan *et al*. ^38^ found that clopidogrel was associated with increased risk of major bleeding compared to ticagrelor. On the other hand, Guan *et al*. ^39^ revealed that ticagrelor was proven to correlate with increased risk of major bleeding compared to clopidogrel. A meta-analysis conducted by Westman *et al*. ^40^ involved 15 papers, consisting of 26,093 patients treated with clopidogrel and 7,192 patients treated with ticagrelor. The authors revealed that although ticagrelor was associated with increased risk of minor bleeding compared to clopidogrel, the incidence of major bleeding was not significantly different between ticagrelor and clopidogrel. Moreover, in the case of fibrinolytic, a meta-analysis involving three RCTs showed that neither ticagrelor nor clopidogrel was correlated with the risk of major bleeding ^41^. Furthermore, in the case of ACS, a meta-analysis involving 10 studies revealed that the risk of bleeding was not significantly different between patients receiving clopidogrel and ticagrelor ^42^. Therefore, it makes sense that in our current meta-analysis, no association was observed between the use of DAPT either clopidogrel or ticagrelor and the risk of major bleeding.

Our findings in sub-group analysis were consistent with our main findings, we emphasized that the incidence of major bleeding either in pre- and post-CABG was not significantly different between clopidogrel and ticagrelor. To our knowledge, until now the major bleeding effect of clopidogrel and ticagrelor in the setting of before and after CABG has not been well defined. Besides the existence of no previous meta-analysis concerning this subject, reports in other case settings did not assess this effect in the pre- or post-intervention context of. Hence, the possible direct and indirect explanations was difficult to clarify. To date, the major bleeding effect of DAPT therapy before and after CABG remained conflicting. A previous study revealed that discontinuation of DAPT therapy 24–72 hours before emergency CABG was proven to increase the risk of major bleeding **** ^35^. Moreover, Deo *et al*. ^43^ also reported that increased risk of major bleeding was observed in post CABG patients treated with ASA and clopidogrel. However, a study by Solo *et al*. ^44^ might support our findings. They evaluated the incidence of major bleeding between ASA and clopidogrel and ASA and ticagrelor. Although statistical analysis was not directly performed, they confirmed that the risk of major bleeding in post CABG patients among different anti-platelets had no strong evidence. Therefore, due to inconclusive reports regarding the risk of major bleeding and DAPT therapy before and after CABG, further studies are required to clarify our current findings.

The theory underlying the risk of major bleeding due to clopidogrel or ticagrelor is not well defined. However, some theories have been proposed. To stimulate inhibition of platelet aggregation, both clopidogrel and ticagrelor are P2Y12 antagonists. However, associated with the risk of major bleeding, each agent has a different mechanism. Clopidogrel is known to irreversibly induce bleeding by inhibiting P2Y12 receptors, and may cause persistent blockade of the adenosine diphosphate (ADP) binding site. Those inhibitory effects may persist until the platelets are renewed in 7–10 days **** ^45^. Therefore, as it has a longer inhibitory effect than ticagrelor, those treated with clopidogrel may be more vulnerable to risk of bleeding than ticagrelor ^42^. Ticagrelor is a reversible P2Y12 receptor antagonist. It works directly on P2Y12 receptors, and therefore may produce rapid inhibition effects and provide rapid recovery of platelet function **** ^46^. It has been already reported that ticagrelor has faster onset and offset than clopidogrel ^47^. As a result, when each drug is stopped, the effect of ticagrelor may disappear faster than clopidogrel. In animal subjects, a study proposed that clopidogrel was found to have 3.5-fold associated with higher bleeding risk compared to ticagrelor ^48^. Clopidogrel is metabolized by cytochrome P2C19 enzyme ^49^, and recent gene-disease interaction studies reported that cytochrome P2C19 CYP2C19*20 C-889T>G (SNP rs11568732) was associated with the risk of bleeding in ACS patients treated with clopidogrel ^50– 52^. Therefore, theoretically, the risk of major bleeding with clopidogrel should be higher than with ticagrelor. However, the evidence from previous large-scale studies, including our present meta-analysis, are conflicting and have not clarified the association. Hence, because it was not supported by the evidence, in our opinion, the risk of major bleeding due to different DAPT, for this time being, might be considered as a hypothesis. In the near future, we expected that more complex study designs might be applied to elucidate the real association between the risk of major bleeding and the use of different DAPT.

To the best of our knowledge, our present study was the first meta-analysis assessing the association between the risk of major bleeding and the use of different DAPT in ACS patients treated with CABG. Our current meta-analysis might clarify the inconclusive findings of previous studies regarding this topic, and we emphasized that clopidogrel, compared to ticagrelor, or vice versa, was not associated with the risk of major bleeding in ACS patients treated with CABG. In the last decade, the use of DAPT, either clopidogrel or ticagrelor, has brought about a dilemma for physicians due to the assumption that one of them was considered to trigger the risk of major bleeding. This dilemma was worsened owing to drug marketing competition among pharmaceutical industries to recommend ticagrelor over clopidogrel. However, our present study indicates that the dilemma was not supported by evidence, and therefore the dilemma might be considered as "the ocean without the waves". The present meta-analysis emphasizes the safety of DAPT administration, either clopidogrel or ticagrelor, in the context of the risk of major bleeding, and hence we expect that our present meta-analysis might reduce the dilemma regarding the risk of major bleeding due to the use of DAPT either clopidogrel or ticagrelor among physicians. The management of ACS patients using CABG has developed in the last decade, and therefore the use of DAPT in CABG management should conform with the adequate evidence. Furthermore, we hope that our current meta-analysis might be involved in the future revision of CABG management for treating patients with ACS.

In our present study, several crucial limitations were observed. First, some factors that might contribute to the risk of major bleeding, such as coagulation factors, history of stroke, chronic kidney disease, hyperglycemia, and anemia ^53^, were not included and controlled for. Second, our current findings should be interpreted with caution due to relatively small sample size. Third, more than a half of our included studies were cross-sectional studies, and might provide the methodological bias. Therefore, our results should be interpreted with caution. In the near future, we expected that further meta-analyses by including papers with higher study design might be conducted to obtain better evidence. Fourth, human factors (skills) were not involved in the analysis. Fifth, other drugs that might govern the risk of bleeding were not analyzed.

## Conclusion

Our meta-analysis reveals that the use of different DAPT either clopidogrel or ticagrelor is not associated with the risk of major bleeding in ACS patients treated with CABG. Our sub-group analysis also fails to confirm this association both in pre- and post-CABG sub-groups. Our findings may provide the clarification of previous conflicting studies in the context of the risk of major bleeding and the use of different DAPT in ACS patients treated with CABG. We also expect that our findings may contribute to the future recommendation of the use of DAPT among ACS patients treated with CABG.

## Data availability

### Underlying data

All data underlying the results are available as part of the article and no additional source data are required.

### Reporting guidelines

Figshare: PRISMA checklist for ‘Comparison of major bleeding in patients with acute coronary syndrome that underwent coronary artery bypass grafting treated with clopidogrel or ticagrelor: a systematic review and meta-analysis’. https://doi.org/10.6084/m9.figshare.11688525.v1 ^22^.
