# Supplementary material for: Comparison of major bleeding in patients with acute coronary syndrome that underwent coronary artery bypass grafting treated with clopidogrel or ticagrelor: a systematic review and meta-analysis
Source: F1000Res. 2020 Sep 7;9:99. Originally published 2020 Feb 10. [Version 2] doi: 10.12688/f1000research.21925.2 (PMC7885293; doi:10.12688/f1000research.21925.2)
Supplement: Peer review history

# Peer review: Reviewer response for version 2

**DOI:** 10.5256/f1000research.29314.r77741

**Published:** 2021-02-15

**Authors:** Mircea Ovanez Balasanian

**Affiliations:** 1 Institute of Cardiovascular Diseases "Prof. dr. George I.M. Georgescu", Iasi, Romania

I consider this systematic review interesting because it shows no significant differences in the risk of major bleeding events of different DAPT use, clopidogrel or ticagrelor, in ACS patients treated with CABG, and also in the pre- and post-CABG settings.

 The rationale and objectives of this review are clearly stated, and the methodology and analysis can be replicated, with appropriate interpretation. The conclusions are supported by the results obtained in this review.

 I think it is a well done work that could serve as a point for further studies in this area.

Are the rationale for, and objectives of, the Systematic Review clearly stated?

Yes

Is the statistical analysis and its interpretation appropriate?

Yes

Are sufficient details of the methods and analysis provided to allow replication by others?

Yes

Are the conclusions drawn adequately supported by the results presented in the review?

Yes

Reviewer Expertise:

Cardiology

I confirm that I have read this submission and believe that I have an appropriate level of expertise to confirm that it is of an acceptable scientific standard.

# Peer review: Reviewer response for version 1

**DOI:** 10.5256/f1000research.24175.r69370

**Published:** 2020-08-25

**Authors:** Sheng-Hu He, Bing Xu

**Affiliations:** 1 Department of Cardiology, Clinical Medical College, Yangzhou University, Northern Jiangsu People's Hospital, Yangzhou, China

This systematic review and meta-analysis aimed to compare the risk of major bleeding between clopidogrel and ticagrelor in ACS patients treated with CABG. It is a meaningful clinical topic that needs more support from evidence-based medicine. According to the results of this study, ticagrelor should be used more confidently in ACS patients treated with CABG.

 However, there are disadvantages in some issues:

1. The characteristic summary of the 13 included trials was not shown in the manuscript. It is an important part of a meta-analysis, which should contain the data of age, gender, race, doses of medication, and bleeding risk scores.
2. Because only 2 of them are RCTs, most of them are case-control trails, the methodological bias of the included studies should be analyzed. There are many methods that can estimate the qualities of the included studies, at least one of them should be used in this study to remind the readers interpreting the results with caution.
3. Although this study has analyzed the main observation, the risk of major bleeding, and did subgroup analysis for pre-surgery and post-surgery patients, other factors should be considered using subgroup analysis, for example, race, region, dose and time, bleeding risk scores if possible.
4. The logic of the discussion is not very clear. The structure of this part should be modified, some points should be simplified, and some duplicate expressions should be removed to make readers understand quicker and easier.

Are the rationale for, and objectives of, the Systematic Review clearly stated?

Yes

Is the statistical analysis and its interpretation appropriate?

Yes

Are sufficient details of the methods and analysis provided to allow replication by others?

Yes

Are the conclusions drawn adequately supported by the results presented in the review?

Yes

Reviewer Expertise:

Interventional cardiology

We confirm that we have read this submission and believe that we have an appropriate level of expertise to confirm that it is of an acceptable scientific standard, however we have significant reservations, as outlined above.

# Response

**Published:** 2020-09-01

**Authors:** Jonny Fajar

**Affiliations:** Brawijaya Internal Medicine Research Center, Department of Internal Medicine, Faculty of Medicine, Universitas Brawijaya, Malang, Indonesia, Indonesia

1. This systematic review and meta-analysis aimed to compare the risk of major bleeding between clopidogrel and ticagrelor in ACS patients treated with CABG. It is a meaningful clinical topic that needs more support from evidence-based medicine. According to the results of this study, ticagrelor should be used more confidently in ACS patients treated with CABG. However, there are disadvantages in some issues: The characteristic summary of the 13 included trials was not shown in the manuscript. It is an important part of a meta-analysis, which should contain the data of age, gender, race, doses of medication, and bleeding risk scores.

**Response: **We have provided the additional characteristics in Table 1.

2. Because only 2 of them are RCTs, most of them are case-control trails, the methodological bias of the included studies should be analyzed. There are many methods that can estimate the qualities of the included studies, at least one of them should be used in this study to remind the readers interpreting the results with caution.

**Response:** The additional information regarding the possibility of methodological bias and to interpret our results with caution has been provided in study limitations.

"Third, more than a half of our included studies were cross-sectional studies, and might provide the methodological bias. Therefore, our results should be interpreted with caution."

3. Although this study has analyzed the main observation, the risk of major bleeding, and did subgroup analysis for pre-surgery and post-surgery patients, other factors should be considered using subgroup analysis, for example, race, region, dose and time, bleeding risk scores if possible.

**Response:** We have tried to perform sub-group analyses in accordance with ethnicity, region, dose, and bleeding risk score; however, the data were imbalanced, and therefore the calculation was unable to be performed.

4. The logic of the discussion is not very clear. The structure of this part should be modified, some points should be simplified, and some duplicate expressions should be removed to make readers understand quicker and easier.

**Response:** We have revised our manuscript in some part to increase the consistency of the text and to avoid the unnecessary repetition without changing the meanings or findings.

# Peer review: Reviewer response for version 1

**DOI:** 10.5256/f1000research.24175.r61678

**Published:** 2020-06-23

**Authors:** Jinesh Bahubali Nagavi

**Affiliations:** 1 Sarada Vilas College of Pharmacy, Mysore, Karnataka, India

- The article is well explained with all the data and results.
- The conclusion is very short and precise.
- Search strategy should be elaborated.
- Objectives should be mentioned clearly in the abstract.
- The discussion part is explained well in detail.
- Statistical analysis and its interpretation is appropriate
- Choice of references is very opt and good.

Are the rationale for, and objectives of, the Systematic Review clearly stated?

Yes

Is the statistical analysis and its interpretation appropriate?

Yes

Are sufficient details of the methods and analysis provided to allow replication by others?

Yes

Are the conclusions drawn adequately supported by the results presented in the review?

Yes

Reviewer Expertise:

Drug interaction studies

I confirm that I have read this submission and believe that I have an appropriate level of expertise to confirm that it is of an acceptable scientific standard.

# Response

**Published:** 2020-09-01

**Authors:** Jonny Fajar

**Affiliations:** Brawijaya Internal Medicine Research Center, Department of Internal Medicine, Faculty of Medicine, Universitas Brawijaya, Malang, Indonesia, Indonesia

Thank you for reviewer comments. We have revised our manuscript in some part to increase the consistency of the text without changing the meanings or findings.

1. The article is well explained with all the data and results.

**Response: **Thank you.

2. The conclusion is very short and precise.

**Response:** Thank you.

3. Search strategy should be elaborated.

**Response:** The search strategy has been provided in methods.

"Search strategy

We conducted a systematic search in PubMed, Embase, Cochrane, and Web of Science up to 20 September 2019. The search strategy, conformed to medical subjects heading (MeSH), involved the use of combination the following keywords: ["Major Bleeding"] AND ["Coronary Artery Bypass Grafting" OR "CABG"] AND ["Dual Anti Platelet Therapy" OR "DAPT"], and ["Clopidogrel" OR "Ticagrelor"]. In our searching strategy, language restrictions were not applied. We only used the study with the larger sample size and that was more up-to-date if we found the same data among studies. Moreover, we also searched the potential papers from the reference list of relevant or eligible studies. We also employed the "related article" option in PubMed to broaden our searching strategy. The potentially relevant papers were identified by two independent investigators (Y.P., M.I.). Disagreement between two independent investigators was resolved by discussion and/or by consulting to the senior investigator (J.K.F.)."

4. Objectives should be mentioned clearly in the abstract.

**Response: **The objective of our study has been outlined in abstract.

"This study aimed to perform a meta-analysis concerning the relation between the risk of major bleeding and the use of different DAPT (clopidogrel or ticagrelor) in ACS patients treated with CABG."

5. The discussion part is explained well in detail.

**Response: **Thank you.

6. Statistical analysis and its interpretation is appropriate.

**Response:** Thank you.

7. Choice of references is very opt and good.

**Response:** Thank you.
